# Association between serum albumin and 42-day postpartum mortality in women with acute fatty liver of pregnancy: a retrospective study

**DOI:** 10.3389/fmed.2025.1574686

**Published:** 2025-06-02

**Authors:** Yuxin Fang, Guangyuan Liao, Xiangbin Li, Yi Fang, Yuanmei Gao

**Affiliations:** ^1^Department of Clinical Medicine, The Third Clinical School of Guangzhou Medical University, Guangzhou, China; ^2^Department of Respiratory and Critical Care Medicine, Key Laboratory for Major Obstetric Diseases of Guangdong Province, The Third Affiliated Hospital of Guangzhou Medical University, Guangzhou, China; ^3^Department of Respiratory and Critical Care Medicine, Key Laboratory for Major Obstetric Diseases of Guangdong Province, Guangdong Provincial Clinical Medical Research Center for Obstetrics and Gynecology, Guangdong-Hong Kong-Macao Greater Bay Area Higher Education Joint Laboratory of Maternal-Fetal Medicine, The Third Affiliated Hospital of Guangzhou Medical University Guangdong Provincial, Guangzhou, China

**Keywords:** serum albumin, acute fatty liver of pregnancy, maternal mortality, maternal complications, fetal outcomes

## Abstract

**Introduction:**

Acute Fatty Liver of Pregnancy (AFLP) is a rare, life-threatening complication during pregnancy, characterized by acute liver failure, endangering both mother and fetus. Albumin (ALB), synthesized by the liver, is vital for maintaining plasma oncotic pressure and transporting substances, acting as a liver function indicator. Given the scarce research on the link between serum albumin levels and adverse outcomes in AFLP, our study aimed to explore the association between serum albumin levels and 42-day postpartum mortality in women with AFLP.

**Methods:**

The study included 139 women with AFLP from the Third Affiliated Hospital of Guangzhou Medical University, from 2010 to 2022. Severe hypoalbuminemia is albumin <25 g/L; patients categorized as ≥25 or <25 g/L. Multivariable Cox proportional hazards regression analyses examined the relationship between serum albumin levels and 42-day postpartum mortality. The main outcome was mortality through 42 days postpartum, with secondary outcomes including maternal complications and fetal outcomes.

**Results:**

Of the participants (average age 30.1 ± 5.4, 60.4% primiparous, the median gestational age was 36.1 ± 3.1 weeks), there were 15 deaths within 42 days postpartum, 10.8% mortality rate. After adjustment, multivariable Cox proportional hazards regression showed that patients with severe hypoalbuminemia faced a markedly higher risk of 42-day postpartum mortality (HR = 5.55, 95% CI 1.5 ~ 20.5). Multiple organ dysfunction and hepatic encephalopathy were more common in the ALB < 25 g/L group. Fetal death and low birth weight were associated with low serum albumin, though not significantly.

**Conclusion:**

Hypoalbuminemia serves as a critical and alterable risk factor for postpartum adverse complications related to AFLP.

## 1 Introduction

Acute fatty liver of pregnancy (AFLP) is a rare, potentially life-threatening condition occurring in the early postpartum phase or the third trimester of pregnancy, which can result in serious maternal and fetal complications including multiorgan failure or even death of the mother and fetus (1). It is currently estimated that there are one to three instances of AFLP for every 10,000 deliveries, indicating a low prevalence among pregnant women (2).

Pathologically, AFLP is characterized by fatty infiltration in the liver (3). During gestation, physiological challenges in the oxidation of long- and medium-chain fatty acids may cause an increase in maternal serum fatty acids, which can be toxic to the liver (4–6). Additionally, enzyme deficits can induce decreased mitochondrial *β*-oxidation of fatty acids, contributing to the development of acute fatty liver during pregnancy. Insufficient levels of this enzyme lead to the buildup of harmful long-chain fatty acid metabolites in the fetus, which can migrate into the mother’s bloodstream, causing maternal liver toxicity, mitochondrial dysfunction, and hepatic failure (7, 8).

Plasma colloid osmotic pressure is regulated in large part by albumin, the most prevalent plasma protein. Other physiological roles of albumin include endothelium stability, hemostatic, anti-inflammatory, and antioxidant actions, solubilization, dissolving, binding, and transporting both endogenous and exogenous chemicals, and capillary permeability modification (9, 10).

Throughout the course of pregnancy, numerous physiological changes may affect the levels of albumin. In the third trimester, plasma volume in pregnant women was increased by 42 to 48% (11). This may lead to hemodilution, known as physiological hemodilution. In this case, although the total amount of albumin may remain unchanged or even increase, the concentration of albumin may decrease. Research has indicated that in healthy pregnancies, serum albumin concentrations decline from a mean of 42 g/L in nonpregnant women to 31 g/L near the end of pregnancy due to an increase in plasma volume (12). Albumin commonly has a lengthy half-life (15–19 days), however, in critically ill individuals, plasma albumin levels can decline by 10–15 g/L in 3 to 5 days (13). According to a prior study, serum albumin experiences structural and functional anomalies in liver damage women, which jeopardizes the protein’s non-oncotic roles as an antioxidant, scavenger, immunological modulator, and endothelial protector. This can lead to a significant decrease in the amount of circulating “effective” albumin (14).

There have been studies quantifying the relationship between other liver and renal function indicators (such as INR, creatine, ALT, etc.) and the poor prognosis of AFLP (15–18); however, no research has yet explored the impact of albumin on mortality and complications in AFLP patients. Currently, whether a decreased albumin can indicate a poor prognosis for women with acute fatty liver of pregnancy (AFLP) remains unknown. According to WHO’s conventional definitions, maternal and pregnancy-related deaths only include those that take place within 42 days after delivery, termination, or abortion (19). Therefore, our study aimed to analyze a large sample of 139 women with AFLP to explore the relationship between serum albumin levels and mortality through 42 days postpartum.

## 2 Materials and methods

### 2.1 Study design and participants

**Figure 1:**
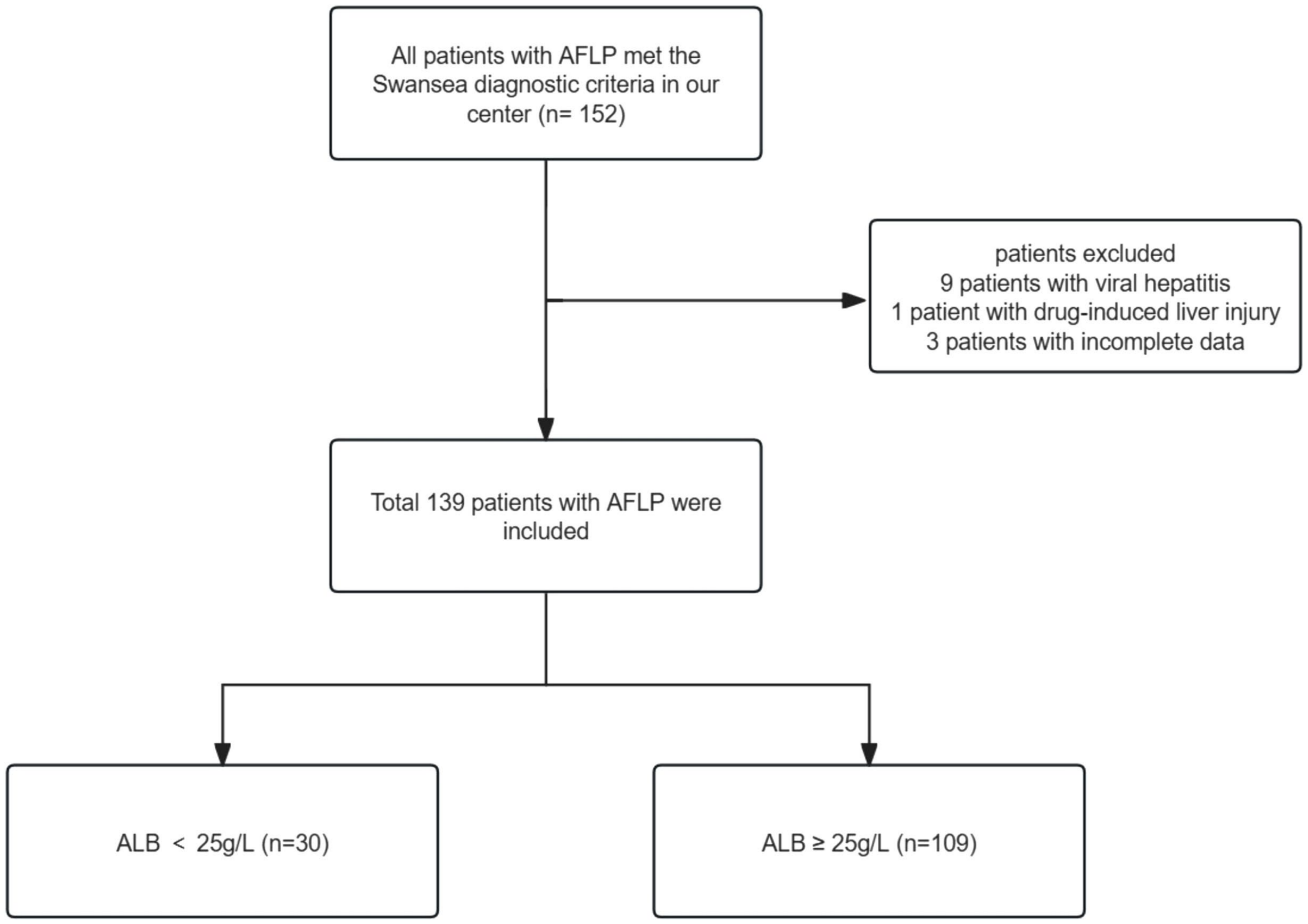
Flowchart of patient selection.

We retrospectively analyzed the data of 139 women who were admitted to the Third Affiliated Hospital of Guangzhou Medical University and diagnosed with AFLP from January 2010 and August 2022. We retrieved AFLP from the medical records system and identified 152 patients who met the criteria. The diagnosis of AFLP was based on the Swansea criteria (20). All women exhibited 6 or more of the Swansea criteria to confirm the diagnosis of AFLP. Upon excluding patients with incomplete data (*n* = 3), viral hepatitis (*n* = 9), and drug-induced liver injury (*n* = 1), we arrived at a final cohort of 139 participants. The patient enrollment workflow is depicted in Figure 1. Given that severe hypoalbuminemia is defined as having an albumin level that is less than 25 g/L (21, 22), we divided patients into two groups based on serum albumin levels: albumin ≥25 g/L (*n* = 109) and albumin <25 g/L (*n* = 30).

The study was approved by the Ethics Committee of the Guangzhou Medical University Third Affiliated Hospital (2,020,087, approved in 2021), following the Declaration of Helsinki (2000) of the World Medical Association. This study followed the Strengthening the Reporting of Observational Studies in Epidemiology (STROBE) reporting guidelines. The severe circumstances, prolonged prothrombin time (PT), and low platelets prevented any of the women from undergoing liver biopsy or liver transplantation. Research has demonstrated that even in the absence of a liver biopsy, no patient would have been overlooked if the Swansea criteria were met (23). Acute kidney injury (AKI) was characterized by serum creatinine (Scr) levels over 90 μmol/L. The diagnosis of disseminated intravascular coagulation (DIC) during pregnancy was established using the DIC score (24). A blood loss of more than 500 mL following vaginal delivery or 1,000 mL following cesarean section delivery within 24 h was referred to as postpartum hemorrhage (PPH) (25). The diagnosis of multiple organ dysfunction syndrome (MODS) and hepatic failure (HE) relies on current guidelines and expert consensus (26, 27).

### 2.2 Collection of clinical data

In this single-center study, a total of 139 women with AFLP were categorized into two groups, according to their serum albumin levels. Given that the level of serum albumin in women may rise after the treatment, all analyses were stratified by serum albumin before treatment. We collected the laboratory data from the first monitoring conducted after the patient’s admission. The laboratory examination results included regular blood tests, hepatic function, kidney functioning, and blood coagulation tests. Serum albumin and other laboratory indicators were determined using a Roche Large Biochemistry Analyzer (cobas c8000).

The primary outcome was mortality through 42 days postpartum, with secondary outcomes including maternal complications and fetal outcomes. All women were followed up for up to 42 days postpartum. Demographic data, such as maternal age, parity, birth mode, anesthetic techniques, gestational age at diagnosis, and interval between symptom start and delivery, were gathered from medical records. Major complications included PPH, MODS, liver failure, HE, and AKI. Fetal outcomes included fetal death, premature delivery, low birth weight, fetal distress, and fetal asphyxia.

The therapies involved plasma exchange, plasma transfusion, antibiotic therapy, and supportive care. In addition, individuals with AFLP commonly face complications such as MODS and hepatorenal syndrome, necessitating artificial liver support therapy and blood purification therapy. Since the long-term effects of liver transplantation are uncertain and acute liver failure from AFLP can resolve on its own within 7 to 10 days following delivery (28), we did not carry out a liver transplant for the patient.

### 2.3 Statistical analysis

Firstly, the data were initially categorized into continuous and categorical variables. Continuous variables were summarized as mean ± standard deviation and compared using Student’s t-test if normally distributed, or as median ± interquartile range (IQR) and compared using the Wilcoxon rank-sum test if not normally distributed. Categorical variables were expressed as percentages and compared using the chi-square test. Baseline and clinical characteristics were compared between participants with albumin levels ≥25 g/L and those with levels <25 g/L. Univariate analysis of risk factors for death was performed using the Cox proportional hazard model, and covariates with *p* ≤ 0.05 were considered significant (to avoid eliminating significant variables). Variables found to be significant in univariate analysis were included in a multivariate Cox regression analysis, performed using a likelihood ratio (LR) approach, *p* ≤ 0.05 (two-tailed) was considered statistically significant.

The Kaplan–Meier method was used for survival analyses across admission ALB levels. We calculated hazard ratios (HRs) and 95% confidence intervals (CIs) through multivariate Cox proportional hazards regression to assess the 42-day postpartum mortality associated with serum albumin. After adjusting for predefined covariates known to be associated with hypoproteinemia, including maternal age, gestational age, parity, red blood cells (RBC), hemoglobin (Hb), activated partial thromboplastin time (APTT), alanine transaminase (ALT), lactic acid (LACT), creatinine (Cr), the mode of delivery, time from onset to termination of pregnancy, plasma exchange, and ICU admission, the results remained robust. We also conducted a smooth curve fitting to explore whether there exists a non-linear relationship between serum albumin and 42-day postpartum mortality. To evaluate the relationship between albumin levels and various secondary outcomes separately, we employed univariate logistic regression analysis.

Every analysis was conducted using the statistical software packages R version 3.3.2^1^ (The R Foundation) and Free Statistics version 1.7 (29).

## 3 Results

### 3.1 Patient selection

152 patients with AFLP met the Swansea diagnostic criteria in our center. Following the exclusion of patients with viral hepatitis (*n* = 9), drug-induced liver injury (*n* = 1), and incomplete data (*n* = 3), the final study cohort consisted of 139 participants. Figure 1 outlines the enrollment process.

### 3.2 Baseline characteristics of the study participants

A total of 139 AFLP women with available data were included in the analysis, and 15 women (10.8%) suffered from death within 42 days postpartum. In all, 109 women (78.42%) had serum albumin levels above 25 g/L while 30 women (21.58%) had values below 25 g/L. There were 15 total deaths (10.8%), with 8 (7.3%) occurring in patients with ALB ≥25 g/L and 7 (23.3%) in patients with ALB < 25 g/L. The mean admission ALB levels were 29.4 ± 5.7 g/L.

Table 1 presents the clinical and biochemical features of the study population, categorized by ALB level (≥ 25 g/L or < 25 g/L). Participants’ mean age ± SD was 30.1 ± 5.4 years, 60.4% were primiparous women, and the median gestational age was 36.1 ± 3.1 weeks. The maternal complications were AKI (*n* = 72, 51.8%), PPH (*n* = 47, 33.8%), MODS (*n* = 26, 18.7%) and HE (*n* = 8, 5.8%). Cesarean section was used in 108 (77.7%) patients. A total of 20 patients (14.4%) underwent plasma exchange (PE). Fetal outcomes include fetal death (*n* = 8, 5.8%), low birth weight (*n* = 64, 47.4%), fetal distress (*n* = 54, 40.6%), and fetal asphyxia (*n* = 48, 36.1%). At baseline, women with severe hypoalbuminemia ALB levels had lower hemoglobin (*p* = 0.001) and red blood cell (*p* = 0.008), and higher lactic acid (*p* = 0.006).

**Table 1:** Clinical characteristics of women with AFLP of the study population according to serum albumin.

Variables	Total (*n* = 139)	ALB<25 g/L (*n* = 30)	ALB ≥25 g/L (*n* = 109)	*p* value
Age, years	30.1 ± 5.4	31.3 ± 4.8	29.7 ± 5.5	0.17
Gestational age	36.1 ± 3.1	35.5 ± 3.2	36.3 ± 3.1	0.187
Parity, *n* (%)				0.956
Unipara	84 (60.4)	18 (60)	66 (60.6)	
Multipara	55 (39.6)	12 (40)	43 (39.4)	
Length of stay, days	9.0 (7.0, 13.5)	9.0 (7.0, 12.0)	8.5 (7.0, 13.8)	0.974
ICU stay time, days	2.0 (0.0, 6.0)	3.0 (0.0, 6.0)	1.0 (0.0, 6.0)	0.505
Time from onset to termination of pregnancy, days	3.0 (1.0, 7.0)	3.0 (2.0, 7.0)	3.0 (1.0, 7.0)	0.732
WBC, (×10^9^/L)	17.3 ± 6.6	18.3 ± 5.3	17.0 ± 6.9	0.349
RBC, (×10^12^/L)	3.5 (2.8, 4.1)	3.0 (2.6, 3.7)	3.6 (3.1, 4.2)	**0.008**
Hb, (g/L)	96.0 ± 25.3	82.9 ± 22.2	99.6 ± 25.0	**0.001**
PLT, (×10^9^/L)	127.1 ± 63.1	125.7 ± 67.9	127.5 ± 62.0	0.89
PT, (s)	20.2 ± 9.8	21.5 ± 8.8	19.9 ± 10.0	0.418
PTA, (%)	51.8 ± 24.8	50.5 ± 29.9	52.1 ± 23.4	0.754
INR	1.8 ± 0.9	1.9 ± 0.8	1.8 ± 0.9	0.392
APTT, (s)	50.2 ± 21.9	50.7 ± 15.6	50.0 ± 23.4	0.887
Fib, (g/L)	1.7 ± 0.9	1.8 ± 1.1	1.7 ± 0.8	0.464
TT, (s)	20.3 (17.8, 23.4)	20.5 (17.6, 24.7)	20.3 (18.0, 23.0)	0.797
AT3, (%)	17.0 (11.0, 23.0)	15.5 (10.0, 18.4)	18.0 (12.0, 24.0)	0.151
D dimer,(ng/mL)	3235.0 (2211.0, 8628.2)	3334.0 (2436.0, 12031.0)	3045.0 (2160.8, 7571.5)	0.378
NH3, (μmol/L)	69.5 ± 35.3	77.2 ± 49.4	67.3 ± 30.1	0.186
ALT, (U/L)	102.0 (40.6, 237.8)	97.9 (57.4, 160.6)	108.5 (36.0, 260.2)	0.898
AST, (U/L)	92.1 (48.2, 238.6)	104.0 (81.0, 170.0)	78.4 (44.4, 247.9)	0.284
TP, g/L	52.1 ± 8.9	44.6 ± 6.9	54.3 ± 8.2	**< 0.001**
ALB, g/L	29.4 ± 5.7	21.6 ± 3.3	31.5 ± 4.2	**< 0.001**
TBil, (μmol/L)	72.0 (39.6, 148.9)	79.8 (46.0, 128.4)	67.9 (39.6, 149.0)	0.91
DBil, (μmol/L)	64.3 (33.7, 119.0)	73.2 (42.7, 113.9)	60.8 (33.2, 119.3)	0.577
GLU, (mmol/L)	5.1 ± 2.1	4.5 ± 1.6	5.3 ± 2.2	0.079
UA, (μmol/L)	451.0 (338.3, 544.5)	432.5 (347.5, 543.5)	455.6 (336.0, 543.0)	0.957
Cr, (μmol/L)	136.1 ± 63.9	154.6 ± 96.6	131.0 ± 50.9	0.073
LACT, (mmol/L)	2.4 (1.8, 3.5)	3.4 (2.4, 4.0)	2.2 (1.7, 3.1)	**0.006**
PCT, (ng/mL)	2.1 (1.1, 4.6)	1.9 (1.2, 4.2)	2.1 (1.1, 4.7)	0.939
NT ProBNP, (pg/mL)	248.2 (87.5, 582.1)	222.9 (98.9, 360.4)	268.1 (86.1, 821.9)	0.278
PPH, *n* (%)				0.213
No	92 (66.2)	17 (56.7)	75 (68.8)	
Yes	47 (33.8)	13 (43.3)	34 (31.2)	
MODS, *n* (%)				**0.02**
No	113 (81.3)	20 (66.7)	93 (85.3)	
Yes	26 (18.7)	10 (33.3)	16 (14.7)	
HE, *n* (%)				**0.012**
No	131 (94.2)	25 (83.3)	106 (97.2)	
Yes	8 (5.8)	5 (16.7)	3 (2.8)	
AKI, *n* (%)				0.066
No	67 (48.2)	10 (33.3)	57 (52.3)	
Yes	72 (51.8)	20 (66.7)	52 (47.7)	
Mode of delivery, *n* (%)				0.101
Transvaginal delivery	31 (22.3)	10 (33.3)	21 (19.3)	
Cesarean section	108 (77.7)	20 (66.7)	88 (80.7)	
Anesthesia method, *n* (%)				0.413
General anesthesia	88 (63.3)	17 (56.7)	71 (65.1)	
Epidural anesthesia	19 (13.7)	3 (10)	16 (14.7)	
No anesthesia	32 (23.0)	10 (33.3)	22 (20.2)	
ICU admission, *n* (%)				0.281
No	40 (28.8)	11 (36.7)	29 (26.6)	
Yes	99 (71.2)	19 (63.3)	80 (73.4)	
Plasma exchange, *n* (%)				1
No	119 (85.6)	26 (86.7)	93 (85.3)	
Yes	20 (14.4)	4 (13.3)	16 (14.7)	
Maternal outcome, *n* (%)				**0.020**
Survival	124 (89.2)	23 (76.7)	101 (92.7)	
Death	15 (10.8)	7 (23.3)	8 (7.3)	
Fetal death, *n* (%)				0.685
No	130 (94.2)	28 (93.3)	102 (94.4)	
Yes	8 (5.8)	2 (6.7)	6 (5.6)	
Premature delivery, *n* (%)				0.058
No	59 (43.1)	8 (27.6)	51 (47.2)	
Yes	78 (56.9)	21 (72.4)	57 (52.8)	
Fetal weight, (g)	2579.2 ± 617.2	2447.2 ± 478.0	2615.3 ± 647.4	0.195
LBW, *n* (%)				0.172
No	71 (52.6)	12 (41.4)	59 (55.7)	
Yes	64 (47.4)	17 (58.6)	47 (44.3)	
Fetal distress, *n* (%)				0.784
No	79 (59.4)	16 (57.1)	63 (60)	
Yes	54 (40.6)	12 (42.9)	42 (40)	
Fetal asphyxia, *n* (%)				0.2
No	85 (63.9)	15 (53.6)	70 (66.7)	
Yes	48 (36.1)	13 (46.4)	35 (33.3)	
Fetal sex, *n* (%)				0.377
Male	91 (66.4)	19 (63.3)	72 (67.3)	
Female	39 (28.5)	8 (26.7)	31 (29)	
Male+Female (twin)	7 (5.1)	3 (10)	4 (3.7)	

Continuous variables are presented as Mean ± SDs or Median (Q1-Q3), categorical variables are presented as numbers (%). Bold values are used to show statistically significant differences between variables.

AKI, Acute kidney injury; ALB, Albumin; ALT, Alanine transaminase; APTT, activated partial thromboplastin time; AST, Aspartate transaminase; AT3, antithrombase III; Cr, creatinine; DBil, Direct bilirubin; Fib, fibrinogen; Glu, blood glucose; Hb, Hemoglobin; HE, hepatic encephalopathy; ICU, intensive care unit; INR, International Normalized Ratio; LACT, lactic acid; LBW, low birth weight; MODS, Multiple organ dysfunction; NH3, blood ammonia; NT-proBNP, N-terminal pro-type B natriuretic peptide; PCT, procalcitonin; PLT, platelet; PPH, postpartum hemorrhage; PT, prothrombin time; PTA, prothrombin activity; RBC, red blood cell count; Tbil, total bilirubin; TT, thrombin time; TP, total protein; UA, uric acid; WBC, white cell count.

### 3.3 Kaplan–Meier survival curve

**Figure 2:**
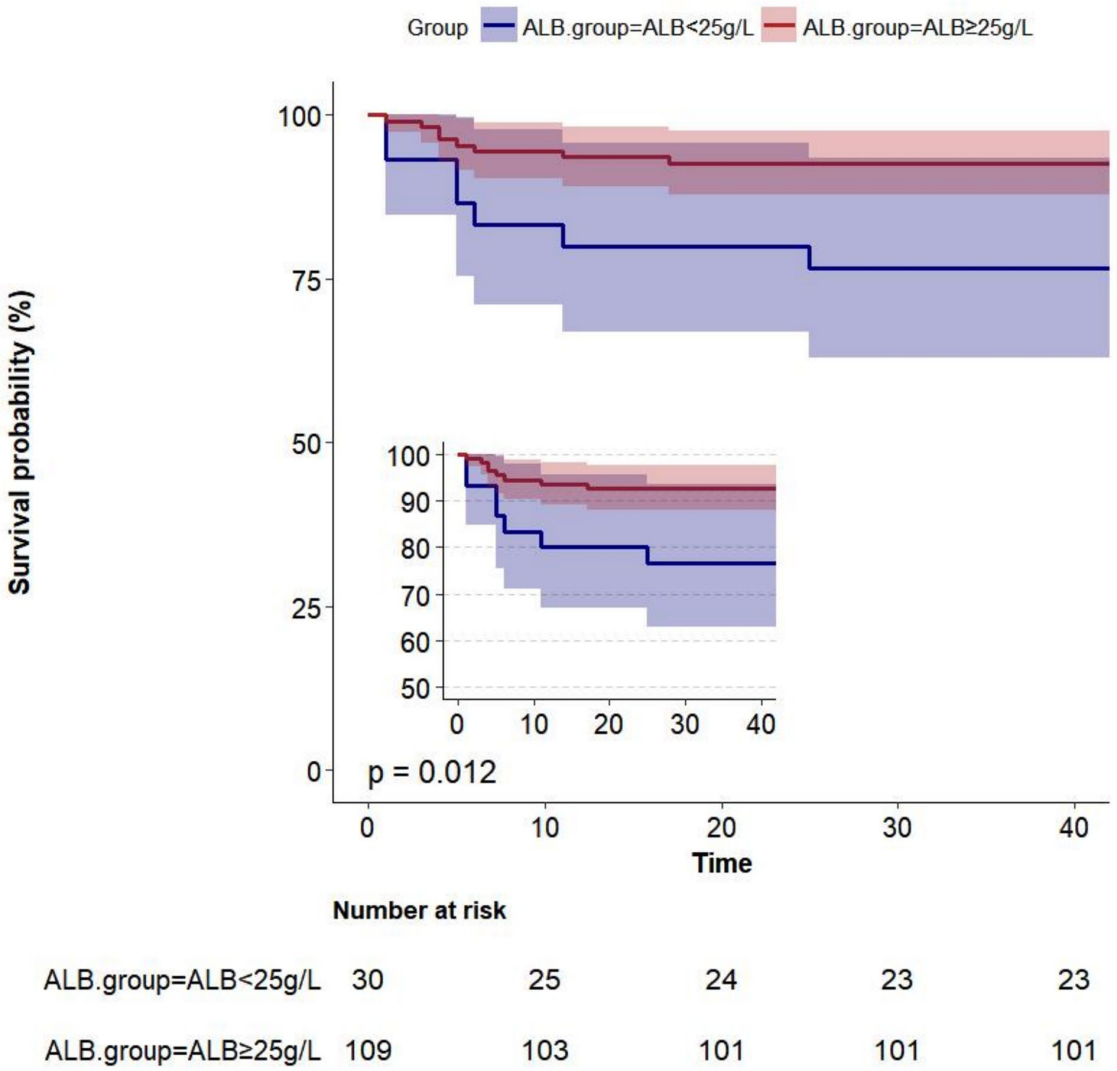
Kaplan–Meier survival curves showing risk of mortality within 42 days postpartum.

Albumin level at admission affected the Kaplan–Meier survival predictions (*p* = 0.012). Within 42 days, 139 women had been followed for postpartum death. At 42 days postpartum, 15 out of 139 women (10.8%) passed away. The Kaplan–Meier predictions of cumulative mortality are shown in Figure 2. The cumulative mortality rate was higher in women with ALB < 25 g/L than in those with ALB ≥ 25 g/L (*p* < 0.05).

### 3.4 Maternal and fetal outcomes

**Figure 3:**
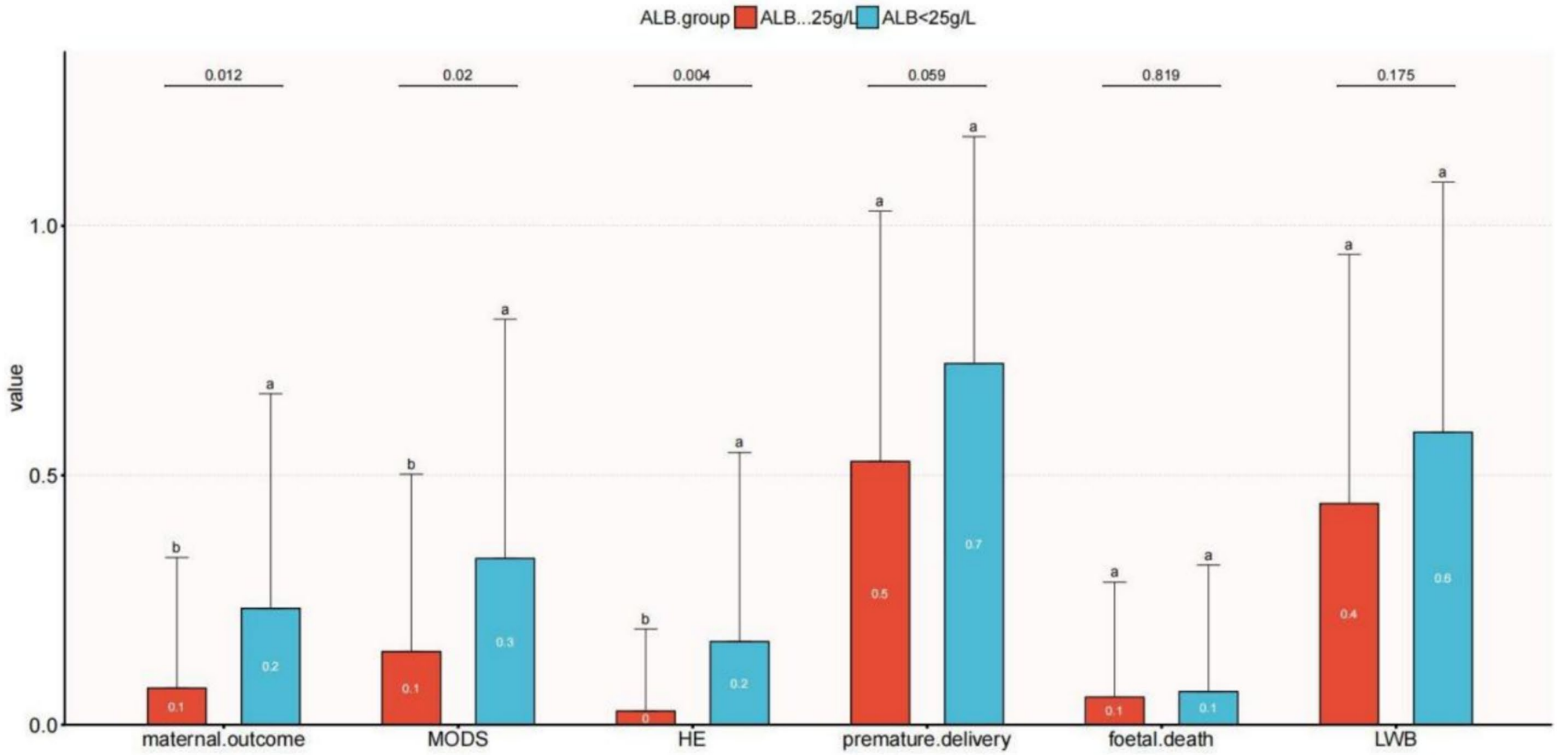
Relationship between serum albumin and clinical outcomes with acute fatty liver of pregnancy. The letters a and b are used to show statistically significant differences between variables. For all variables with the same letter, the difference between the means is not statistically significant. If two variables have different letters, they are significantly different. HE, hepatic encephalopathy; LBW, low-birth-weight infant; MODS, multiple organ dysfunction.

Univariate logistic regression analysis was used to evaluate the relationship between serum albumin (ALB) levels and secondary outcomes (LBW, HE, MODS, fetal death, and premature delivery), as illustrated in Figure 3. MODS was more common in the ALB <25 g/L group than in the ALB ≥25 g/L group (*p* = 0.02). Additionally, compared to women with ALB<25 g/L, individuals in the ALB < 25 g/L group had a greater level of HE (*p* = 0.012). Fetal mortality and low birth weight were associated to low serum albumin, though not significantly.

### 3.5 Association between albumin levels and postpartum mortality

Findings from the multivariable Cox proportional hazards regression for the association between serum albumin levels and 42-day postpartum mortality are shown in Table 2. When albumin was analyzed as a continuous variable, a significant independent negative association was discovered between serum albumin and 42-day postpartum mortality in the non-adjusted crude model (HR: 0.88, 95% CI: 0.81–0.96; *p* = 0.005); meanwhile, further adjustment did not significantly affect the results. Model I was adjusted for sociodemographic variables (age, gestational age, parity). Model II was further adjusted for red blood cells (RBC), hemoglobin (Hb), activated partial thromboplastin time (APTT), alanine transaminase (ALT), lactic acid (LACT), and creatinine (Cr). Model III was further adjusted for the mode of delivery, time from onset to termination of pregnancy, plasma exchange, and ICU admission. After adjusting for all covariates that are potentially connected to low serum albumin, the results remained robust. We can see from Table 2, above 25 g/L, for every1 g/L increase in serum albumin, there was a 19% decrease in 42-day postpartum mortality (adjusted HR = 0.81; 95% CI 0.71 ~ 0.92, Model III, Table 2).

**Table 2:** Association between serum albumin levels and mortality through 42 days postpartum in multivariable Cox proportional hazards regression analyses.

Variable	Total	Event (%)	Unadjusted		Model I		Model II		Model III	
			HR (95% CI)	*p* value	HR (95% CI)	*p* value	HR (95% CI)	*p* value	HR (95% CI)	*p* value
Continuous ALB per 1 g/L	140	15 (10.7)	0.88 (0.81 ~ 0.96)	0.005	0.88 (0.81 ~ 0.97)	0.008	0.85 (0.75 ~ 0.95)	0.006	0.81 (0.71 ~ 0.92)	0.002
ALB≥25 g/L	109	8 (7.3)	1 (Ref)		1 (Ref)		1 (Ref)		1 (Ref)	
ALB<25 g/L	30	7 (23.3)	3.43 (1.24 ~ 9.46)	0.017	3.21 (1.15 ~ 8.97)	0.026	4.72 (1.39 ~ 15.98)	0.013	5.55 (1.5 ~ 20.5)	0.01

Model 1: Adjust for Age+gestational age+parity; Model 2: Adjust for Model 1 + RBC+Hb+APTT+ALT+LACT+Cr; Model 3: Adjust for Model 2 + Mode of delivery+Time from onset to termination of pregnancy +Plasma exchange+ICU admission.

ALB, albumin; ALT, alanine transaminase; APTT, activated partial thromboplastin time; CI, confidence interval; Cr, creatinine; Hb, hemoglobin; HR, hazard ratio; RBC, red blood cell count; Ref: reference.

After adjusting for all variables, the multivariable Cox proportional hazards regression indicated that women with an ALB < 25 g/L had a 4.55-fold higher risk of 42-day postpartum mortality (HR = 5.55, 95% CI 1.5 ~ 20.5, Model III, Table 2) than women with an ALB ≥ 25 g/L.

### 3.6 Curve fitting for linear association

Finally, non-linear relationships between serum albumin and 42-day maternal postpartum mortality were examined using a smooth curve fitting technique (Supplementary Figure S1). Serum albumin levels and 42-day postpartum mortality had a negative association after all potential confounders were taken into account (nonlinearity: *p* = 0.856).

## 4 Discussion

To our knowledge, this study provided the first assessment of 42-day postpartum mortality associations with serum albumin based on a large sample of 139 women between 2010 and 2022. The Swansea standards serve as a recognized international benchmark for the diagnosis of acute fatty liver of pregnancy (AFLP). While the guidelines do not address albumin in the Swansea standards, we found that women with serum albumin levels below 25 g/L had a significantly higher risk of 42-day postpartum mortality compared to those with albumin levels of 25 g/L or higher, thus highlighting the critical need for careful monitoring of albumin pregnant women with AFLP. Additionally, MODS and HE were more commonly observed in the group with severe hypoalbuminemia. These results emphasize the importance of monitoring serum albumin as a predictor of postpartum complications.

Up to now, research on AFLP has primarily concentrated on exploring risk factors. Nulliparity, male fetal sex, and multifetal gestation are potential risk factors of AFLP (4, 8, 30, 31). These factors, however, are unchangeable and static. Peng et al. reported that INR, total bilirubin, fibrinogen, and low platelet were confirmed as risk factors for AFLP-related three-month mortality (15). The days between the onset of symptoms and hospitalization and parturition were the high-risk factors for AFLP fatality, according to a prior study that Li reported (18). According to Gao et al. (32), serum creatinine, total bilirubin, and abortion history are all separate risk factors for maternal death. Chen et al.’s (33) study revealed a correlation between the postpartum recovery duration of AFLP and the levels of platelets, total protein, and total bilirubin. While liver and kidney function indicators and coagulation indicators have received considerable attention in AFLP risk factor studies, albumin, which is a vital liver function indicator, remains overlooked.

Although serum albumin has not been considered an independent risk factor for the prognosis of women with AFLP (15–17, 34), hypoalbuminemia was frequently observed in patients suffering from AFLP. A recent study found that albumin levels in 88% of the women with AFLP had dropped to less than 30 g/L (35). In another study, abnormal liver, renal, and coagulation tests, as well as extremely low serum albumin, were seen in the laboratory results of 91–100% of women with AFLP (36).

Decreased hepatic synthesis causes a progressive decrease in serum albumin, which affects colloid osmotic pressure (37). In the early stages of MODS, fluid retention, and systemic tissue edema are caused by increased permeability of systemic capillaries to water and protein, and decreased plasma colloidal osmotic pressure (38). A reduction in osmotic pressure linked to hypoalbuminemia can lead to a deficiency in intravascular fluid, thereby impairing organ perfusion and potentially causing multiple organ dysfunction syndrome (39). This helps to clarify why MODS are more prevalent in the group with severe hypoalbuminemia (ALB < 25 g/L).

According to a cohort study of 298 individuals, the most serious and potentially fatal maternal consequences are hepatic encephalopathy and postpartum hemorrhage (18). Hepatic encephalopathy can span from mild cases to severe ones, frequently accompanied by elevated intracranial pressure (40, 41). And mild hepatic encephalopathy can sometimes go unnoticed if ammonia (NH3) is not monitored effectively. Our study found that individuals with ALB < 25 g/L were more susceptible to hepatic encephalopathy. Hence, clinicians should be vigilant regarding the risk of hepatic encephalopathy in those with lower albumin levels. Monitoring in an intensive care unit is necessary for patients with lower albumin, along with careful assessment of their neurological condition and encephalopathy severity.

Despite considering various important factors, we cannot establish a causal relationship, and serum albumin might be a predictive factor rather than a direct cause of health. However, it remains crucial to prioritize primary prevention of albumin levels falling beyond the physiological range during pregnancy due to its significant effect on various maternal and offspring outcomes. Furthermore, the goal of treatment is to assist the liver in returning to normal function, which includes avoiding potentially hepatotoxic chemicals and providing adequate nutritional support. In this process, a gradual rise of serum albumin levels can be seen as a positive sign of restored liver function and improved prognosis.

Plasma exchange has become a focal point for treating AFLP. Endotoxin elimination, coagulation factor support, intravascular volume and albumin, electrolyte regulation, and acid–base balance are the suggested advantageous mechanisms (42). Combining plasma exchange and plasma perfusion may result in better survival outcomes than conventional treatment by itself (2).

Multidisciplinary collaboration is required for the management of women with AFLP. Early identification and intervention, including close collaboration between hepatologists, obstetricians, dietitians, and other professionals, provides comprehensive care for people with AFLP. Serum albumin levels should be closely monitored as part of this collaborative effort. A comprehensive treatment strategy, including nutritional support and close monitoring of serum albumin levels, can optimize maternal and fetal health, reduce mortality, and improve maternal and fetal prognosis.

There are various limitations on this study. First, there was only a limited number of women included in this study because of the low incidence of AFLP. Even yet, our sample size was greater than that of other comparable research (15–17, 33, 34, 43, 44). Future multicenter collaborations are needed to validate these findings in larger sample sizes of prospectively enrolled populations. Furthermore, because this was a single-center trial, bias was unavoidable. Additionally, the lack of follow-up information post-discharge restricted our focus to short-term postpartum outcomes, rather than examining the lasting effects of hypoalbuminemia. We acknowledge that the 12-year study period may introduce temporal bias, though the core management strategy—timely pregnancy termination for this condition—remained unchanged in institutional guidelines during the entire 12-year period.

Taken together, we show that severe hypoalbuminemia has procedure-specific effects and calls for targeted management strategies to enhance maternal outcomes. In the absence of definitive guidelines, our findings encourage obstetricians to measure albumin levels and treat hypoalbuminemia appropriately.

## Data Availability

The datasets presented in this article are not readily available because the datasets analyzed in this study are not publicly available due to their inclusion in an ongoing research project, but can be obtained from the corresponding author upon reasonable request. Requests to access the datasets should be directed to YG yuanmeigao@126.com.

## Footnotes

^1^ http://www.Rproject.org
