# Supplementary figures and images for: Association between serum albumin and 42-day postpartum mortality in women with acute fatty liver of pregnancy: a retrospective study
Source: Front Med (Lausanne). 2025 Jun 2;12:1574686. doi: 10.3389/fmed.2025.1574686 (PMC12171446; doi:10.3389/fmed.2025.1574686)

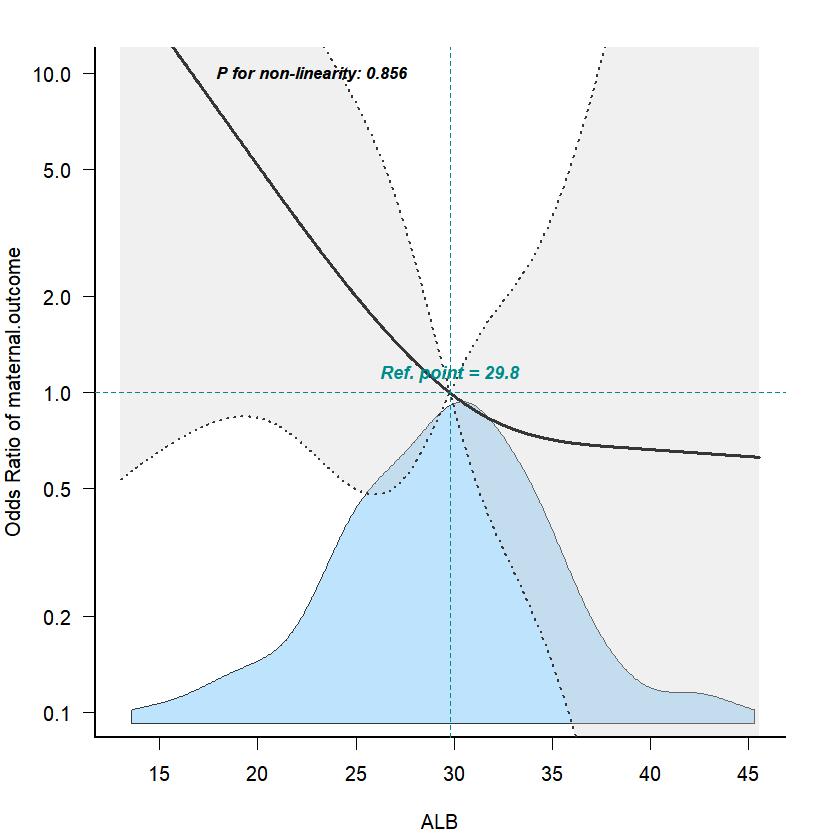

Supplement: Supplementary file 1 [file Image_1.JPEG]
